# 

**Published:** 1856-07-01

**Authors:** 


*** *The "Psychological Retrospect" cannot, owing to unavoidable circumstances, appear this Quarter. The Editor makes amends for this omission by appending nearly three extra sheets of matter to this number.*

